# Immune drivers of physiological and pathological pain

**DOI:** 10.1084/jem.20221687

**Published:** 2024-04-12

**Authors:** Aakanksha Jain, Sara Hakim, Clifford J. Woolf

**Affiliations:** 1 F.M. Kirby Neurobiology Center, Boston Children’s Hospital, Boston, MA, USA; 2 Department of Neurobiology, Harvard Medical School, Boston, MA, USA

## Abstract

Physiological pain serves as a warning of exposure to danger and prompts us to withdraw from noxious stimuli to prevent tissue damage. Pain can also alert us of an infection or organ dysfunction and aids in locating such malfunction. However, there are instances where pain is purely pathological, such as unresolved pain following an inflammation or injury to the nervous system, and this can be debilitating and persistent. We now appreciate that immune cells are integral to both physiological and pathological pain, and that pain, in consequence, is not strictly a neuronal phenomenon. Here, we discuss recent findings on how immune cells in the skin, nerve, dorsal root ganglia, and spinal cord interact with somatosensory neurons to mediate pain. We also discuss how both innate and adaptive immune cells, by releasing various ligands and mediators, contribute to the initiation, modulation, persistence, or resolution of various modalities of pain. Finally, we propose that the neuroimmune axis is an attractive target for pain treatment, but the challenges in objectively quantifying pain preclinically, variable sex differences in pain presentation, as well as adverse outcomes associated with immune system modulation, all need to be considered in the development of immunotherapies against pain.

## Teaser

Immune cells instruct the development and persistence of adaptive and maladaptive pain states by modifying neural activity in the end organs, nerves, dorsal root ganglia, spinal cord, and brain, therefore opening the neuroimmune axis as a target to treat pain.

## Introduction

There are several quite distinct types of pain, some physiological and others pathological, each with different mechanisms, dynamics, and clinical manifestations. Nociceptive pain is the unpleasant acute sensation we experience when we encounter a potentially damaging noxious stimulus in our environment, such as a pinprick, contact with something too hot, or acute exposure to a chemical irritant like capsaicin, the pungent ingredient in chili peppers. This pain is triggered by specialized high-threshold sensory neurons called nociceptors. Nociceptors express specific transducer channels that convert noxious stimuli into electrical activity and then transmit the resulting evoked action potentials into specific nociceptive circuits in the spinal cord and brain that lead to both acute flexion reflexes and the perception of pain. This pain is a key protective mechanism against potential danger, both by initiating an immediate withdrawal response from the damaging stimulus and by teaching us to avoid such stimuli by virtue of the unpleasantness of the sensory experience.

Upon actual damage to tissue after a traumatic injury, surgery, or infection, the initial immediate nociceptive pain changes to a longer-lasting pain due to the inflammation induced by immune cells. We begin to both feel spontaneous pain and experience mechanical and thermal tenderness at the site of the inflammation (primary hypersensitivity) (Perl et al., 1976) and even beyond, in surrounding non-inflamed tissues (secondary hypersensitivity) (LaMotte et al., 1991). Inflammatory pain hypersensitivity can be adaptive, as it helps us guard the injured part of the body from further damage while healing occurs or an infection is overcome. However, inflammatory pain can also become maladaptive and debilitating, as in the case of autoimmune disorders like rheumatoid arthritis, where the pain can persist even in the absence of ongoing clinical inflammation (Jurczak et al., 2022).

Neuropathic pain, which occurs after an injury to the nervous system, is always maladaptive. Here, the sensation of pain can be evoked by normally innocuous stimuli (allodynia) (Decosterd and Woolf, 2000), is amplified and prolonged in response to a noxious stimulus (hyperalgesia) (Decosterd and Woolf, 2000), and occurs in the absence of any stimulus (spontaneous pain) (Zheng et al., 2022). Neuropathic pain is the consequence of persistent pathological changes in the functioning of the damaged nervous system. Finally, there are clinical pain syndromes, currently referred to as nociplastic or “dysfunctional” pain, that occur in the absence of a noxious stimulus, inflammation, or nervous system damage (Kosek et al., 2016). This pain arises from the pathological malfunctioning of the intact nociceptive system due to abnormal excitability or connectivity and causes syndromes with persistent localized or widespread pain, like fibromyalgia (Bułdyś et al., 2023), which do not have a clear peripheral pathological trigger nor a central lesion.

Traditionally, the generation and perception of both physiological and clinical pain were thought to primarily reflect only neuronal activity; however, it has become increasingly clear that almost all forms of pain occur as a result of, or are modified by, close interactions between the immune and somatosensory systems (Costigan et al., 2009a; Ghasemlou et al., 2015; Tanaka et al., 2023). Essentially, both systems have been adaptively designed by evolution to detect danger and both contribute to tissue homeostasis as a key protective mechanism but also interact in disease states to generate ongoing pathological clinical pain.

Nociceptor terminals in peripheral tissues, such as the skin, gut, and joints, are detectors of key aspects of the tissue microenvironment, especially the presence of any potentially damaging external or internal stimulus. Immune cells work in parallel with these sensory neurons to constantly survey tissues. However, this dual damage detection process is not independent, the immune cells are adjacent to and interact with sensory neurons directly and reciprocally by complex receptor–ligand interactions in infections (Baral et al., 2018; Chiu et al., 2013), allergies (Perner et al., 2020; Serhan et al., 2019; Talbot et al., 2015), malignancies (Balood et al., 2022), and tissue damage (Hoeffel et al., 2021). Consequently, we need to see neuroimmune interactions as a combined and integrated driver and modulator of pain and must in consequence appreciate that pain is a neuroimmune condition.

The peripheral nerve is also a site of immune involvement in pain. A diverse population of immune cells is present within healthy intact peripheral nerves and many more are recruited and activated after nerve damage, inflammation, or neuropathy, and their ligands can locally modify axonal function. The cell bodies of nociceptor sensory neurons reside in the dorsal root ganglia (DRG). These cell bodies can undergo long-term changes in function by transcriptional plasticity, which can be initiated directly by immune cells present in the DRGs. The central axons of DRG neurons project to the dorsal horn of the spinal cord, which is the first location of nociceptive circuits in the central nervous system (CNS) (Todd et al., 2002, 2005). The spinal cord and brain also rely on immune signals from microglia for homeostasis and normal function (Ji and Suter, 2007). Immune cells contribute to the triggering both of acute and persistent pain, either by directly interacting with neurons in the peripheral nervous system or the CNS or by producing systemic immune mediators that enter the CNS and alter its function. Therefore, pain is, to a substantial extent, an expression of immune-neuronal disturbances that occur throughout the pain circuitry from the periphery to the cortex.

Here, we review recent studies that reveal the key role of diverse immune cell types in different types of pain generation that highlight the complex mechanisms by which the immune system regulates nociceptive, inflammatory, neuropathic, and nociplastic pain, and that indicate why the neuroimmune axis is emerging as a major potential target to treat or prevent pain.

## Immune control of acute nociception

**Figure 1.**
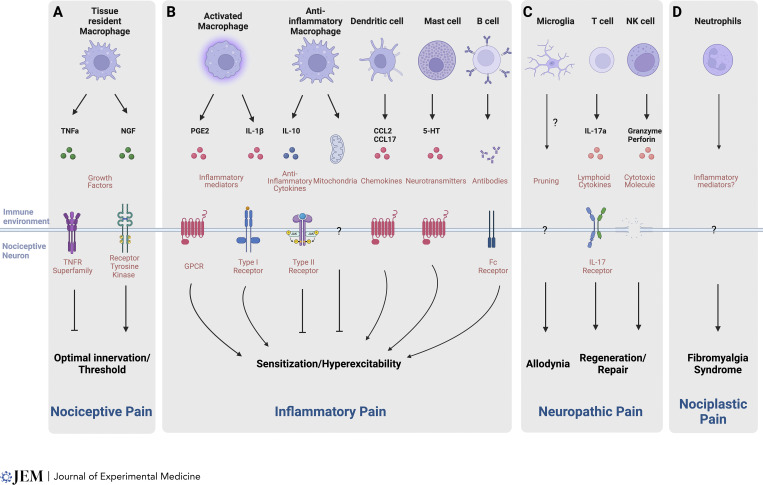
**Immune activity regulates various aspects of DRG neuron function in healthy and disease states. (A–D)** Immune cell types, effector molecules, and receptor classes are involved in (A) nociceptive, (B) inflammatory, (C) neuropathic, and (D) nociplastic pain. NK, natural killer.

Nociceptive pain is the immediate sensory perceptual response to an acute noxious challenge. Since this occurs within milliseconds, it has long been thought to be purely a neural response, independent of any immune involvement. However, it was recently shown that the threshold for activation of nociceptors, even at a steady state, is set by immune-derived ligands (Fig. 1 A). Nerve growth factor (NGF) produced by dermal macrophages has a tonic action on nociceptor terminals in the skin (Tanaka et al., 2023). In the absence of this persistent basal NGF activity, mice show a blunted response to noxious stimuli, demonstrating that tonic immune cues instruct acute nociception. Immune cells also modify tissue innervation by nociceptors during development, thereby contributing to the establishment of baseline sensitivity to noxious stimuli. An absence of the receptor for TNFα signaling in sensory neurons results in axon overgrowth in the skin and increased sensitivity to NGF, which consequently manifests as lower pain thresholds (Wheeler et al., 2014). Both innate and adaptive immune cells produce TNFα and are likely to contribute to regulating the growth and refinement of nociceptor terminals in peripheral tissues. These findings lay the foundation for future studies to uncover the exact nature and extent of the homeostatic interactions that exist between tissue-resident immune cells and the peripheral terminals of the nociceptors in different tissues under healthy conditions.

## Innate and adaptive immune mechanisms of inflammatory pain

The peripheral sensitization of nociceptors is a key driver of inflammatory pain hypersensitivity (Chen et al., 1999). This is the direct result of a reduction in the threshold of activation of transducer receptors and ion channels on nociceptor peripheral terminals in response to the presence of inflammation (Chuang et al., 2001). Immune mediators, including cytokines (Binshtok AM et al., 2008; Guptarak et al., 2013), chemokines (Cunha et al., 1991; Zhang et al., 2005), and antibodies (Andoh and Kuraishi, 2004; Jurczak et al., 2022), produced during inflammation can directly act on receptors expressed on nociceptor terminals, causing nociceptor sensitization through several mechanisms, including posttranslational modifications (Hudmon et al., 2008; Masuoka et al., 2020) and altered membrane trafficking (Higerd-Rusli et al., 2023; Kopach et al., 2013) (Fig. 1 B).

### Effects of tissue inflammation on nerve terminals

The immune microenvironment is diverse and dynamic, consisting of both tissue-resident and infiltrating immune cells. Neutrophils are the first responders to tissue infection and injury; however, their depletion does not alter acute pain hypersensitivity (Ghasemlou et al., 2015). Tissue-resident immune cells, such as dendritic cells and macrophages, do however, mediate acute inflammatory pain hypersensitivity (Ghasemlou et al., 2015; Silva et al., 2022). After a skin incision, resident dendritic cells produce the chemokines CCL2 and CCL17, triggering neuronal hyperexcitability via the CCR4 receptor expressed on DRG neurons. IL-1β, TNFα, IL-6, PGE2, and reactive oxygen species are also pro-nociceptive mediators generated by macrophages in inflamed tissues (Chen et al., 2020). IL-1β, TNFα, and IL-6 also contribute to long-term pain by modulating synaptic plasticity in the dorsal horn neurons (Kawasaki et al., 2008). Mast cells are also a resident myeloid population in the skin that promotes inflammatory pain after a skin incision via a BH4-dependent synthesis of serotonin (Starkl et al., 2023, *Preprint*). This mechanism might also contribute to the inflammatory itch that occurs during wound healing. Interestingly, macrophages can also be anti-nociceptive as macrophage depletion delays recovery from zymosan-induced inflammatory pain, a model of pathogen-induced inflammation (Bang et al., 2018). This dual effect is due to the functionally plastic nature of macrophages. In the initial phase of injury, macrophages produce inflammatory cytokines that directly act on nociceptor terminals via cognate receptors (Locati et al., 2020). However, during the resolution phase of the injury, macrophages acquire an anti-inflammatory program, producing pain-reducing cytokines like IL-10 (Locati et al., 2020). Recently, we identified a macrophage-derived protein, thrombospondin 1, which competitively alleviates prostaglandin-induced nociceptor sensitization via inhibition of protein kinase A activity (Jain et al., 2023, *Preprint*). Interferon receptor signaling in nociceptors also controls nociceptor sensitization both at a steady state and in a bone cancer pain model (Donnelly et al., 2021). Nociceptors produce type 1 interferons in response to a nociceptor-specific stimulator of interferon genes (STING) agonism (Donnelly et al., 2021). STING-induced type 1 interferon also inhibits human and monkey nociceptor excitability in vitro, which is attributed to reduced Ca^2+^ and Na^+^ currents (Donnelly et al., 2021). However, systemic interferon production induced by polyI:C injection in mice leads to increased pain hypersensitivity via mitogen-activated protein kinase interacting protein kinases–mediated eIF4E-dependent mechanisms (Barragán-Iglesias et al., 2020). This underscores how the context of an insult dictates the neuroimmune mechanisms of pain induction. While posttranslational modifications downstream of kinase activation are a major driver of nociceptor sensitization (Hudmon et al., 2008; Masuoka et al., 2020), the inflammatory milieu also activates other signaling pathways, including mTORC2, which promotes long-term nociceptor sensitization by enhancing nociceptor terminal branching (Wong et al., 2022). While the immune driver of mTORC2 activity in nociceptors is not yet known, these findings highlight that the immune microenvironment in tissues acts as a rheostat to finely tune inflammatory pain hypersensitivity by enhancing or suppressing peripheral sensitization as well as inducing structural changes in nociceptors. While prosensitizing immune mediators have been studied extensively, an unexpected pain-suppressing role of immune cells in inflammation is also now becoming clear.

### DRG immune cells: Messengers of peripheral inflammation

Single-cell transcriptomic studies have revealed diverse immune populations in the DRG, suggesting that neuroimmune interactions in the sensory ganglia may regulate pain remotely from the site of injury (Renthal et al., 2020). Macrophages residing at the blood–DRG barrier constantly monitor the vasculature and sample macromolecules from the blood, providing a route for communication between the periphery and DRG-resident immune cells during inflammation (Lund et al., 2024). Like their function in peripheral tissues, macrophages located in DRGs can both promote and suppress pain. In a mouse model of osteoarthritis, nitric oxide–producing inflammatory macrophages infiltrate into the DRG and contribute to persistent pain (Raoof et al., 2021), while reparative macrophages can help resolve pain by promoting recovery of oxidative phosphorylation in sensory neurons (van der Vlist et al., 2022). These studies reveal an immune regulation in DRGs, which opens questions about the exact nature and function of immune interactions within DRGs, both at steady state and during peripheral inflammation and after nerve injury, and its consequences.

### Neuronal sensing of systemic immune changes

In addition to myeloid cells, adaptive immune cells, including T and B cells, can also regulate pain hypersensitivity. Either a genetic or antibody-mediated neutralization of the T cell cytokine, IL-17a, alleviates mechanical hyperalgesia in antigen-induced arthritis (Ebbinghaus et al., 2017; Pinto et al., 2010), while IL-35 produced by regulatory T cells alleviates pain behavior in a mouse model of multiple sclerosis (Duffy et al., 2019). T cells are also mediators of endogenous opioid-induced analgesia during pregnancy (Rosen et al., 2019). However, it is unclear if T cells act directly on sensory neurons or if their role is via an action on other immune cells. It will be interesting to investigate whether there is a sensory neuronal antigen that recruits and activates T cells, and if immunological memory plays a specific role in chronic inflammatory pain. Peripheral nociceptor terminals express Fc receptors for antibodies produced by B cells, and IgE production following ingestion of food allergens drives visceral pain in a mast cell–dependent fashion (Aguilera-Lizarraga et al., 2021). Interestingly, the injection of antibodies from rheumatoid arthritis patients into mice results in mechanical hypersensitivity in the absence of measurable inflammation (Wang et al., 2019), and this pain-related behavior in mice can be alleviated by morphine but not by anti-inflammatory analgesics (Jurczak et al., 2022). While this implies that pain can occur without clinical inflammation (Wigerblad et al., 2016), in patients autoantibody generation requires initial activation of innate immunity and T cells. Therefore, in chronic inflammatory diseases like rheumatoid arthritis, the early targeting of immune drivers that act on the nociceptive system could be helpful in preventing the development of persistent maladaptive pain. These findings open potential novel therapeutic modalities for inflammatory pain beyond the standard approach of reducing prostaglandin production by cyclooxygenase 2 inhibition using non-steroidal anti-inflammatory agents.

## Immune regulation of neuropathic pain from the periphery to the cortex

Neuropathic pain is caused by an injury to, or a lesion of, the nervous system. This includes both direct mechanical injury, for example following an amputation, and more complex etiologies, such as exposure to damaging chemicals (e.g., chemotherapy-induced peripheral neuropathy or CIPN) and metabolic diseases like diabetes (Costigan et al., 2009b). In diseases with a systemic inflammatory component, such as obesity or autoimmunity, it is conceivable that immune cells may infiltrate into peripheral nerves and alter ion channel composition, neuronal excitability, and myelination, contributing to a neuropathic pain-like phenotype, even in the absence of actual physical injury to sensory axons. The mechanisms of neuropathic pain are highly complex and involve neurons in the periphery (Lyu et al., 2000; Sukhotinsky et al., 2004) as well as in the spinal cord (Woolf, 1983; Woolf et al., 1988–1989) and higher-order regions of the brain (Liu et al., 2018; Wager et al., 2013). Increasing data indicate that immune cells contribute to the initiation and maintenance of neuropathic pain (Fig. 1 C).

### Nerve-associated immune cells: Sensors and protectors of peripheral nerve health

Peripheral nerves harbor a diverse repertoire of immune cells, both at steady state and following nerve injury, as can now be extensively evaluated by single-cell sequencing (Kalinski et al., 2020). Transcriptional profiles of macrophages in peripheral nerves are well characterized in healthy and injured nerves (Ydens et al., 2020), but their role in sensory dysfunction associated with peripheral neuropathy is not known. Recently, we have shown that macrophages infiltrate the peripheral nerves in diabetic mice and protect against axon degeneration and heat hypoalgesia (Hakim et al., 2024, *Preprint*). In addition to directly acting on neurons, nerve macrophages can also modulate non-immune cell functions. Myeloid cell–derived Gas6 acts on Schwann cells to promote their maturation after peripheral nerve injury (Stratton et al., 2018). While the effects of this macrophage–Schwann cell interaction specifically on pain were not evaluated, this study implies that immune–glial interactions in nerves may modify axonal properties of sensory neurons and in this way alter their pain-triggering actions. Natural killer cells are another cell type recruited to the site of nerve injury, where they promote regeneration of injured afferents and prevent post-injury pain hypersensitivity (Davies et al., 2019). While several studies point to a neuroprotective role for immune cells in peripheral nerves, macrophages also expand in nerves after a melanoma inoculation in response to an m-Csf1 signal from Schwann cells, and this contributes to an element of melanoma cancer pain dependent on Transient receptor potential ankyrin 1 (TRPA1) (De Logu et al., 2021). What remains to be determined is which signals from injured or diabetic nerves promote a neuroprotective immune response and how to leverage those signals to prevent further injury, promote regeneration, and alleviate neuropathic pain.

### Immune response to chronic neuropathic insults in the DRG

Residing outside the blood–brain barrier, DRGs are prone to exposure to systemic cues as well as immune infiltration. For example, CX3CR1^+^ macrophages in DRGs proliferate and expand in response to peripheral nerve injury in mice, and ablation experiments show that DRG macrophages both initiate and sustain mechanical allodynia following injury (Yu et al., 2020). In addition, macrophage numbers increase in the DRG during CIPN in rats, and this contributes to mechanical allodynia in a TLR4- and MCP-1–dependent manner (Luo et al., 2019). The T cell cytokine IL-17a directly activates TRPV1^+^ nociceptors, and this contributes to the generation of mechanical allodynia in a mouse model of CIPN (Luo et al., 2019). Mounting evidence also points to CD8^+^ T cells in the DRG as major players in neuropathic pain. In CIPN, CD8^+^ T cells in DRGs help resolve neuropathic pain, and as a consequence, their deletion prevents its resolution (Laumet et al., 2019). CD8^+^ T cells signal to macrophages via IL-13 to promote the release of the cytokine IL10, which resolves pain hypersensitivity (Singh et al., 2022). To leverage the sensory neuron–T cell axis to alleviate pain, it is critical to reveal the antigen specificity and functional profile of T cells responding to neuropathic insults. Current tumor immunotherapy research focusing on the activation of T cells could potentially aid the development of T cell–based pain treatments.

### Advancements and challenges of defining CNS neuroimmune axis in pain

Immune cells also reside in and infiltrate into the spinal cord and contribute there to neuropathic pain. Macrophages act as gatekeepers preventing excessive neuroinflammation in the spinal cord and in this way reduce the persistence of neuropathic pain (Niehaus et al., 2021; Tansley et al., 2022b). Microglia, the resident myeloid cells in the CNS, have a major role, at least in mouse models of neuropathic pain. While crucial for repair and remyelination after spinal cord injury (Li et al., 2020; Wang et al., 2020), microglia also proliferate and are activated after peripheral nerve injury via signaling through the colony-stimulating factor released from injured neurons that acts on its receptor CSF1R expressed by microglia, which contributes to the generation of pain (Guan et al., 2016) (Fig. 1 C). Spinal microglia can prune inhibitory interneuron synapses, which contributes to persistent mechanical allodynia by an excitatory: inhibitory imbalance (Yousefpour et al., 2023). In addition to pruning, microglia degrade the perineuronal nets that enwrap spino-parabrachial neurons in lamina I of the dorsal horn, which also contributes to the generation of pain-related behavior after nerve injury (Tansley et al., 2022a). Much like peripheral myeloid cells, microglia adopt various states depending on their local milieu, and understanding what changes they undergo in those conditions that initiate chronic neuropathic pain will be critical for uncovering the specific microglial pathways involved and the downstream consequences of this for pathological nociceptive circuit dysfunction. Because microglia are heavily involved in neurodegenerative diseases, it is conceivable that immune cells residing in the brain may also contribute to chronic pain. For example, after peripheral nerve injury, microglia in the primary somatosensory cortex upregulate brain-derived neurotrophic factor (BDNF), and deletion of BDNF in CX3CR1^+^ microglia, as well as selective ablation of S1 microglia, alleviates nerve injury–induced mechanical hypersensitivity (Huang et al., 2021). Similarly, peripheral nerve injury causes increased TNF and BDNF signaling in microglia in the spinal cord and hippocampus, which produces synaptic alterations that contribute to neuropathic pain and memory deficits after nerve injury (Liu et al., 2017).

Chemotherapeutic agents that cause peripheral neuropathy can also produce a cognitive impairment termed “chemo brain,” which is modulated by microglia in the brain (Tang et al., 2022). Alterations in cortical circuitry or cortical microglia could, therefore, contribute to pain in patients with peripheral neuropathy. Additionally, it is conceivable that abnormal neuronal activity in those regions of the brain involved in the higher-order levels of somatosensory processing or in the affective components of pain modify microglia, which in turn might alter neuronal activity and contribute to abnormal nociceptive circuit function. Imaging studies on chronic pain patients show increased glial activation in the thalamus and somatosensory cortices (Loggia et al., 2015).

Microglia are clearly important players in pain regulation; however, their diverse functionalities make it challenging to understand their mechanistic role and to target them for therapeutic purposes. While it is possible to specifically deplete or isolate microglia using Cre drivers, a lack of genetic and other tools to precisely alter the specific functions of microglia has impaired a comprehensive understanding of their involvement in pain. For example, minocycline, a commonly used tool to pharmacologically inhibit microglial activation, only suppresses the inflammatory function of microglia (Hammond et al., 2019; Kwon et al., 2022). Deletion of the *Trem2* gene in human microglia disrupts their phagocytic function but does not affect cytokine expression, as based on transcriptomic data (McQuade et al., 2020). The contextual role of microglia was also demonstrated in regard to microglial-dependent neuropathology in the anterior singular cortex in a mouse model of intestinal inflammation. Deletion of the microglial receptor TREM-1 mitigated visceral hyperpathia in the acute inflammation while a TREM-2 deficiency attenuated depression-like symptoms during remission (Wu et al., 2023). More comprehensive studies are needed to determine if and how immune cells, specifically microglia, in the CNS change during injury or peripheral neuropathy, and whether, in consequence, they contribute to the presence or persistence of neuropathic pain.

## Does nociplastic pain have an immune component?

Nociplastic or dysfunctional pain occurs in the absence of peripheral inflammation or damage to the nervous system and includes a number of inexplicable pain conditions, including complex regional pain syndrome (CRPS), fibromyalgia, and irritable bowel syndrome (Kosek et al., 2016). The molecular and cellular underpinnings of these dysfunctional pain syndromes are unknown since they cannot be directly explained by either peripheral or central pathological drivers. However, transferring antibodies from fibromyalgia syndrome (FMS) patients into healthy mice triggers non-localized hypersensitivity and tenderness (Goebel et al., 2021). In a mouse model of chronic widespread pain, neutrophils infiltrate into DRGs, and their depletion abolishes the establishment of chronic pain–associated behavior (Caxaria et al., 2023) (Fig. 1 D). Moreover, neutrophils from FMS patients also confer pain in mice (Caxaria et al., 2023). What is not clear, though, is what are the underlying immune-derived effector molecules and where and how they interact with pain-processing sensory neurons. In a tibial fracture and casting mouse model of CRPS, TLR4 in microglia was shown to be important in early pain progression in males more than females, but important in the persistence of this pain in both sexes (Huck et al., 2021). In addition, in the same tibial fracture model, depleting microglia is followed by microglial repopulation of the spinal cord, and it is the repopulated microglia that leads to the pain resolution rather than the depletion of the microglia (Donovan et al., 2024). It remains to be determined what effector molecules secreted by the repopulating microglia are responsible for this effect. In addition to changes in the spinal cord, systemic inflammation can cause the brain to initiate non-localized pain. High levels of C-reactive proteins during infancy are associated with spinal cord hypersensitivity and cortical nociceptive processing in response to normally innocuous tactile stimuli, even after the inflammation has long resolved, suggesting that an inflammatory event in the periphery can induce long-term changes in pain-processing/nociceptive neurons within the brain (Cobo et al., 2022). The recognition of an immune cell involvement in the development of nociplastic pain prompts fundamental questions of which immune cells are interacting with nociceptive neurons, in which anatomical compartment, and if and how they may trigger or facilitate chronic pain syndromes.

## Targeting the neuroimmune axis to treat pain

The diverse anatomical locations, disease states, and modalities where different immune cells contribute to pain render the immune system attractive for potential therapeutic intervention. Current analgesic treatments have multiple undesirable outcomes, including substance abuse disorders, sedation, nausea, pruritis, and reduced cognitive function. Non-steroidal anti-inflammatory analgesic drugs block prostaglandin production by immune cells during inflammation and are effective in acute inflammatory pain conditions. However, chronic non-steroidal anti-inflammatory drug (NSAID) use can damage the gastric lining and impact cardiac function. Several novel aspects of the neuroimmune interface are emerging as promising targets for developing safer and more effective analgesics. Immunotherapies to block IL-1, TNFα, and IL-6 are currently used to treat autoimmune conditions; however, these might also be effective in treating all those pain conditions driven by these cytokines. In a small clinical study, systemic or local blockade of IL-6 effectively attenuated low back pain, highlighting how targeting the immune system could be a promising starting point for discovering novel neuroimmune-acting analgesic drugs (Ohtori et al., 2012; Sainoh et al., 2022). An inducible T cell costimulator agonist antibody that is currently being developed for clinical use to promote anti-tumor T cell immunity resolved mechanical hypersensitivity in mouse models of CIPN, pointing to the possibility of immunotherapy-CIPN treatments (Sankaranarayanan et al., 2023). In addition, immune signatures could prove to be useful biomarkers for pain and analgesia (Russo et al., 2020). In patients with chronic low back pain, for example, higher inflammatory responses triggered by transient neutrophils are associated with protection against pain (Parisien et al., 2022) (Fig. 1 D).

### Consideration for sex differences

A critical aspect of designing effective novel therapeutics for pain is the consideration of sex differences in pain perception, processing, and memory. Several pain syndromes only occur in women, including endometriosis and menstrual pain. Women also make up the majority of patients with debilitating pain conditions like FMS and osteoarthritis (Clauw, 2014; Tschon et al., 2021). Many explanations for sex differences in pain have been proposed, including hormonal modulation, differences between X and Y chromosomes, and distinct gender-dependent midbrain–brainstem connectivity (Mogil, 2012). Neuroimmune interactions are also emerging as promising candidates to explain at least some sex differences in pain. Females produce higher levels of inflammatory mediators, which not only makes them more susceptible to various autoimmune diseases but might also result in increased inflammatory pain (Billi et al., 2019). The pain-enhancing enzyme prostaglandin D2 synthase is present at increased levels in females, suggesting a potential sex difference in prostaglandin production by immune cells (Shen et al., 2023). IL-17a–induced mechanical allodynia in a mouse model of CIPN requires the presence of estrogen receptors on neurons, and in consequence, only occurs in female mice (Luo et al., 2021). After nerve injury, microglia are responsible for mechanical allodynia in male mice while in females they are not (Sorge et al., 2015). An analysis of neuroimmune signaling pathways in DRGs from patients has revealed that there are also distinct cytokine signaling pathways associated with neuropathic pain in males and females (Ray et al., 2023). Activating TLR4 signaling evokes a higher production of pain-promoting cytokines and chemokines in microglia derived from males than female mice (Agalave et al., 2014). However, not all aspects of microglial-dependent pain mechanisms show sexual dimorphism as discussed in the nociplastic pain section (Huck et al., 2021). Optogenetically activating microglia leads to similar neuronal activation and pain behavior in male and female mice (Yi et al., 2021). The TMEM16F and TRPV4 ion channels expressed by microglia lead to microglial activation and promote neuropathic pain in both sexes (Batti et al., 2016; Hu et al., 2023). Microglia have versatile functions, including phagocytosis (Fourgeaud et al., 2016; Marín-Teva et al., 2004; Sierra et al., 2010), cytokine secretion (Clark et al., 2015; Shigemoto-Mogami et al., 2014), and antigen presentation (Beauvillain et al., 2008; Herz et al., 2015). The contrasting sex-dependent effects of microglia could result from the diverse mechanisms by which microglia interact with pain-triggering neuronal circuits. Sex differences also underlie the relative efficacy of certain analgesics. Female mice need more opioids to achieve analgesia than males, and this reduced sensitivity is dependent on the presence of T cells (Rosen et al., 2019). These studies warrant that investigation into the immune mechanisms of pain must include sex as a key biological variable to help develop effective analgesic therapeutic approaches for pain conditions in all individuals.

### Developing neuroimmune-based analgesics: A double-edged sword

The immune system has emerged as a prominent therapeutic target for many chronic and previously intractable ailments, including cancer, and neurodegenerative and neurodevelopmental diseases (Hammond et al., 2019; Kwon et al., 2022). However, immunotherapies are potentially a double-edged sword. Long-term inhibition of immune activity results in immunosuppression, rendering patients susceptible to infections (Souto et al., 2014; Zhang et al., 2017). On the other hand, using NSAIDs to suppress acute inflammatory neutrophil response correlated with the development of chronic low back pain in patients (Parisien et al., 2022). Immune activation, therefore, must be carefully modulated to prevent potentially serious complications from cytokine release syndromes and autoinflammatory conditions while allowing for protective immune activity against chronic pain development (Morris et al., 2022).

## Challenges and advances in the preclinical detection of pain

To develop effective therapies targeting those elements of the neuroimmune axis that drive pain, it is imperative to have robust and reliable preclinical pain metrics. Pain is a complex biopsychosocial phenomenon that can only be comprehensively and directly investigated in those humans who can report the presence of a subjective pain sensation. However, failure to be able to report the pain does not mean it is not there, as in newborn babies or adults who have a speech impediment, and we, therefore, need, even in humans, to be able to identify pain-related changes in the nervous system such as functional magnetic resonance imaging measurements or behavior (crying/grimacing) as indirect surrogate measures of pain and its relief with analgesics. Preclinical measures of pain are all indirect, but this does not mean the pain is not there, it just cannot be directly reported. Historically, we have relied mainly on reflex-based methodologies in rodent models as a proxy for pain, such as elicitation of a withdrawal response to a noxious stimulus. While this enables advances in pain research, it remains challenging to quantify and ascertain the existence and nature of clinical pain conditions in rodents.

Recently, we have made advances in the study of pain-like behaviors in rodents. Using an automated detection technology, we are now able to objectively quantify mechanical paw pain sensitivity in rodents by tracking both paw position and the force of contact in an investigator-independent manner using machine learning, providing a reliable readout for the presence of nociceptive or inflammatory pain (Jones et al., 2020). In addition, the grimace scale can be used to assess a rodent’s discomfort as another measure of pain or of an aversive experience (Langford et al., 2010). By recording freely moving mice in the dark, we can evaluate exactly the degree, force, and timing of paw contact with the surface as a proxy for pain-induced avoidance. We can also record and extract whole-body posture that can inform us about behavioral features that cannot be detected by manual scoring (Zhang et al., 2022). This now allows us to identify behavioral features that, if reversed by known analgesics, can be deemed as pain-like features in mice. Continued development of machine learning tools for the identification of pain biomarkers in preclinical models will be key to the assessment of neuroimmune targeting candidates that have the highest likelihood of translating into successful therapies in the clinic. Machine learning may also impact assessing the phenotype of pain patients and help identify who is more susceptible to severe persistent pain.

## Conclusion

We are beginning to increasingly appreciate that immune cells play a major role at almost every location in pain circuits, from sensory nerve endings in the periphery all the way to the cortex. It is now clear that to fully understand acute, inflammatory, neuropathic, and nociplastic pain mechanisms, we need to thoroughly investigate the nature, timing, and extent of immune involvement in nociceptive circuitry in health and disease states. A more granular understanding of both innate and adaptive immune cell states that can be targeted to treat pain is needed to assess whether current modalities and approaches in immunotherapy could be leveraged for more effective treatments of inflammatory, neuropathic, and chronic pain conditions, or whether novel approaches are required. Essentially, we would like to argue, based on the substantial recent literature, that it is not possible or appropriate to separate the immune and nervous systems in pain; they act together to produce pain and need to be studied together to aid the development of more effective and safer pain treatments.
